# Incidence and prevalence of salivary gland tumours in Valparaiso, Chile

**DOI:** 10.4317/medoral.20337

**Published:** 2015-06-02

**Authors:** Juan Araya, René Martinez, Sven Niklander, Maureen Marshall, Alfredo Esguep

**Affiliations:** 1 Universidad Andres Bello. Departamento de Patología y Cirugía Oral. Facultad de Odontología, Viña del Mar, Chile

## Abstract

**Background:**

To determine the incidence and prevalence of salivary gland tumours in the province of Valparaíso, Chile.

**Material and Methods:**

Retrospective review of salivary gland tumours diagnosed between the years 2000 and 2011 from four local pathology services. Information on demographics and histopathology were retrieved from the medical records.

**Results:**

The study sample consisted of 279 salivary gland tumours. Prevalence and incidence rates per 100.000 persons were 15.4 and 2.51, respectively. Most of the neoplasms corresponded to benign tumours (70.3%). The most affected gland was the parotid gland. Pleomorphic adenoma was the most common benign tumour (53.8%) and mucoepidermoid carcinoma was the most common malignant tumour (7.2%).

**Conclusions:**

Salivary gland tumours are uncommon neoplasms that usually arise in the parotid gland. Pleomorphic adenoma and mucoepidermoid carcinoma were the most common benign and malignant tumours reported in this series.

**Key words:**Salivary gland tumours, benign tumours, malignant tumours, salivary glands neoplasms, cancer, neoplasia.

## Introduction

Salivary gland tumours represent an uncommon heterogeneous group of neoplasms with complex clinicopathological characteristics (1). The prevalence of these tumours varies between studies, but has been estimated to be 3-6% of all head and neck tumours (2).

Tumours of many different origins can arise in the salivary glands. Some authors suggest that the histopathology of these neoplasms is more complex and diverse than any other site in the body (3). The World Health Organisation (WHO) proposed the first histological classification of salivary gland tumours in 1972 (4). Due to advances in the understanding of the aetiology and behaviour of these tumours as well as their wide morphological diversity, the WHO published the third and last edition of this classification in 2005 (5).

The glands most commonly affected are the parotid and submandibular glands respectively, usually by benign tumours (6). When the minor salivary glands are affected, it is usually by malignant tumours and almost every tumour arising from the sublingual gland is malignant (3).

Many studies have been performed in order to describe the epidemiology of benign and malignant salivary gland tumours (1-3,6,7). The incidence, prevalence, age, gender, anatomical distribution and survival rates varies between different parts of the world (3,6) and is not necessarily representative of the population of Chile. To our knowledge there is only one study published in the literature performed in a Chilean population but this study included a considerable smaller sample size and was conducted in another area of the country.

It is important to note that this is the first study that characterises patients diagnosed with salivary gland tumours in the province of Valparaiso-Chile, presenting information on the incidence and prevalence in one of the most important geographical areas of Chile.

## Material and Methods

Data from salivary gland tumours diagnosed between the years 2000 and 2011 was collected from four local Oral and General Pathology services (Hospital Dr. Gustavo Fricke, Hospital Carlos van Buren, Hospital Naval NEF and the Histopathology Laboratory of the Dentistry Faculty of Universidad Andrés Bello). Informed consent was obtained from each patient. In case the patient had died, a close relative was contacted in order to obtain informed consent. Reference populations for each of the centres was recorded in order to calculate prevalence and incidence rates. All four centres together have an estimated population of 1.3645.000.

Cases were included if the diagnosis was confirmed histopathologically and where age, gender and site of the tumour were recorded in the medical records. All cases were classified under the histological criteria suggested by the WHO in 2005 (5). Tumours diagnosed using the 1991 WHO classification criteria for salivary gland tumours were revised by an expert pathologist and assigned a new diagnosis based on the 2005 WHO classification criteria.

Cases were excluded if the histopathological diagnosis, gender, age and/or site of the tumour were missing. Squamous cell carcinomas and non-epithelial tumours arising in minor salivary glands were excluded because of the uncertainty of the origin of those tumours.

The chi-square test was used for the comparison of qualitative variables. A (*P*-value of 0.05 or less was considered significant.

Ethical approval was sought and obtained from the ethical committee of the Dentistry Faculty of Universidad Andres Bello.

## Results

Between the years 2000 and 2011, 279 salivary gland tumours were diagnosed in the province of Valparaiso. The prevalence was 15.24 per 100.000 and the incidence was 2.51 per 100.000.

Forty one point nine percent (n= 117) of the tumours occurred in men and 58.1% (n= 162) occurred in women. One hundred and ninety six tumours (70.3%) were benign and 83 (29.7%) were malignant. Of the benign tumours, 80 (40.8%) occurred in men and 116 (59.2%) occurred in women. Of the malignant tumours, 37 (44.6%) occurred in men and 46 (55.4%) in women. The mean age for benign and malignant tumours was 53.3 ± 19.09 and 60.9 ± 26.6 years respectively. Two hundred fifty nine tumours (92.8%) were primary epithelial neoplasms, 19 (6.8%) were primary non-epithelial neoplasms and only one tumour (0.4%) was a metastasis.

- Primary epithelial tumours:

The distribution of the primary epithelial tumours (n=259) is shown in  Table 1. The parotid gland was the most commonly affected site (n=151) followed by the submandibular gland (n=49) and the palatal salivary glands (n=29). There were 188 (72.6%) benign epithelial tumours and 71 (27.4%) malignant epithelial tumours. The majority of the tumours diagnosed in the major salivary glands were benign neoplasms with a benign-malignant ratio of 3.9:1. The benign-malignant ratio in the minor salivary glands was 1:1.

**Table 1:**
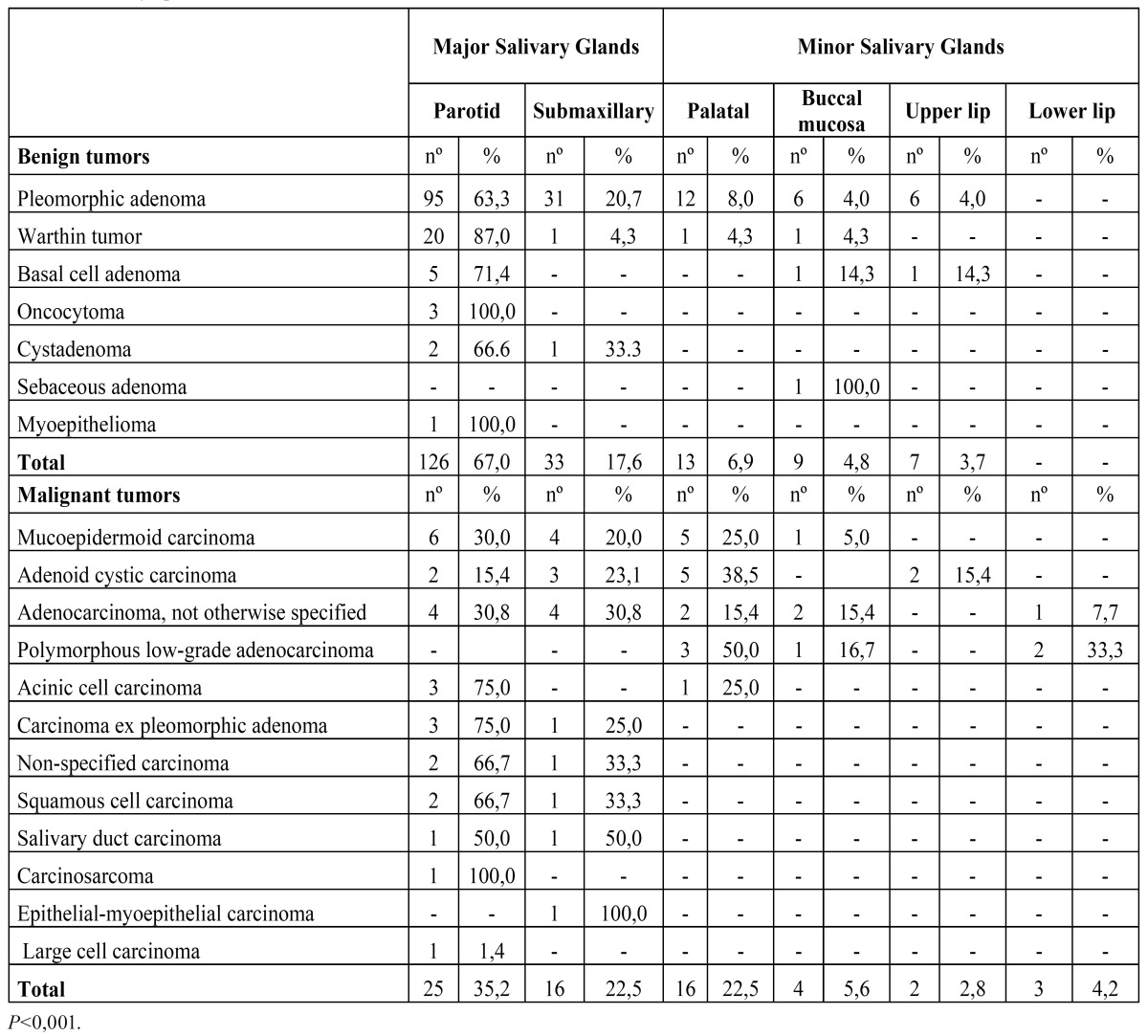
Primary epithelial tumours.

**Figure 1:**
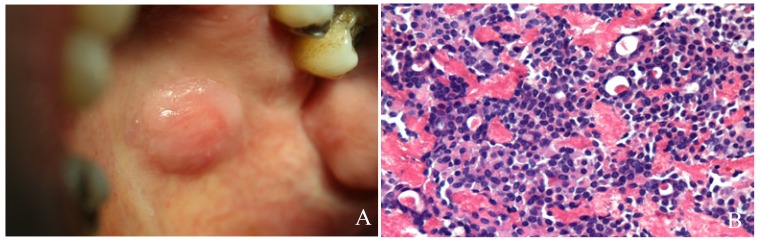
Pleomorphic adenoma: A.Well defined, round to ovoid tumour located in the left side of the hard palate. B. Proliferation of epithelial and myoepithelial cells, some with plasmacytoid appearance, forming duct-like structures containig eosinophilic secretory material with a mesenchimal myxoid component (HE, 10x).

**Figure 2:**
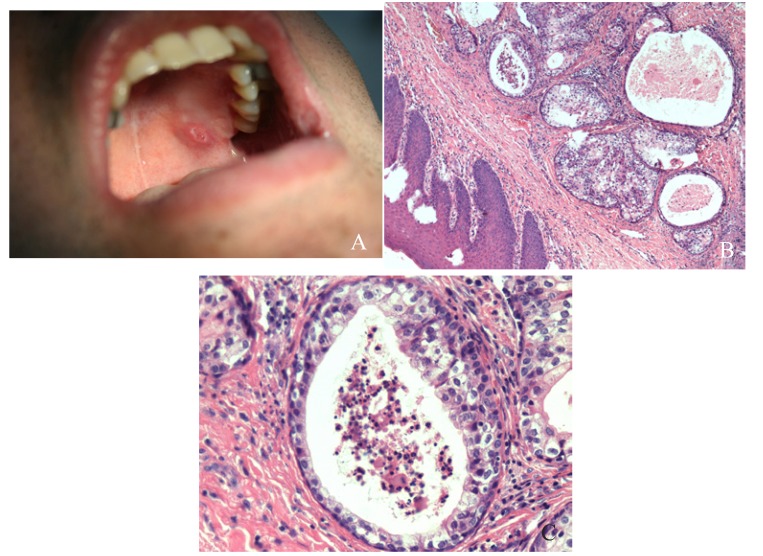
Mucoepidermoid carcinoma: A. Ill-defined, ulcerated, erythematous mass located on the left hard palate. B: Sheets of squamous and mucous producing cells forming cystic spaces. (HE 10x) C: Cystic space linned by numerous, large, mucous producing cells. (HE; 40x).

**Figure 3:**
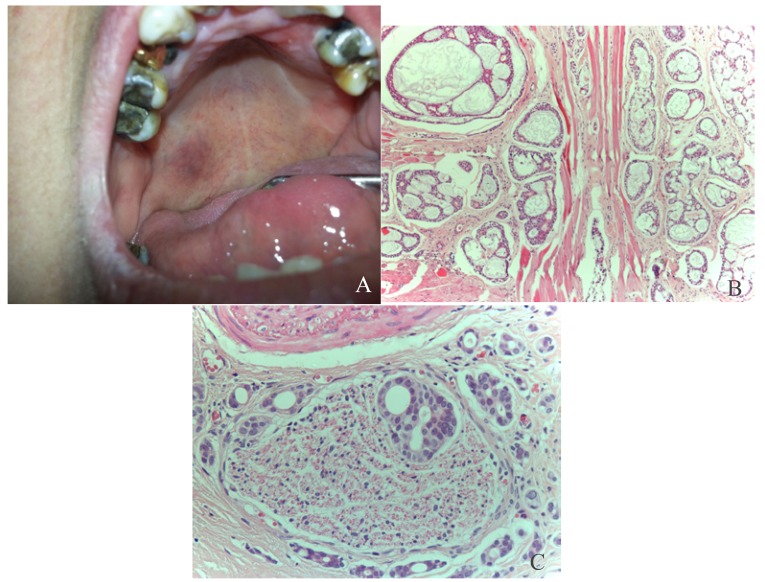
Adenoid cystic carcinoma: A. Ill-defined mass on the right side of the hard palate with an erythematous surface. B: Adenoid cystic carcinoma, cribiform pattern: proliferation of ductal and miopepithelial cells with hyperchromatic nuclei, arranged in nests forming microcystic spaces filled with basophilic mucoid material, separated by hyalinized fibrous conective tissue. (HE, 10x) 3. C: Perineural invasion, a common feature of adenoid cysic carcinoma (HE, 40x).

The pleomorphic adenoma (Fig. 1) was the most common tumour with a total of 150 cases, representing 53.8% of all tumours (epithelial and non-epithelial) and 57.9% of the epithelial tumours. The Warthin tumor was the second most common tumour with 23 cases (8.2%). Both lesions showed a predilection for the parotid gland, which was statistically significant when the different locations of these two tumours (*p*<0.001) was compared. There were no cases of epithelial tumours affecting the sublingual glands. The mucoepidermoid carcinoma (Fig. 2) and the adenoid cystic carcinoma (Fig. 3) were the third and fourth most prevalent lesions respectively. The mucoepidermoid carcinoma affected the major and minor salivary glands evenly, while the majority of the adenoid cystic carcinomas were found to affect the minor salivary glands.

The distribution of epithelial tumours by gender is shown in  Table 2. The male to female ratio was 1:1.35. The male to female ratio for benign and malignant tumours was 1:1.41 and 1:1.22 respectively. The majority of the pleomorphic adenomas were found in women (91 of the 150 tumours), which was statistically significant (*p*=0.009). Sixteen of the 23 cases of Warthin tumour were found in men, but this difference was not found to be statistically significant (*p*= 0.061).

**Table 2:**
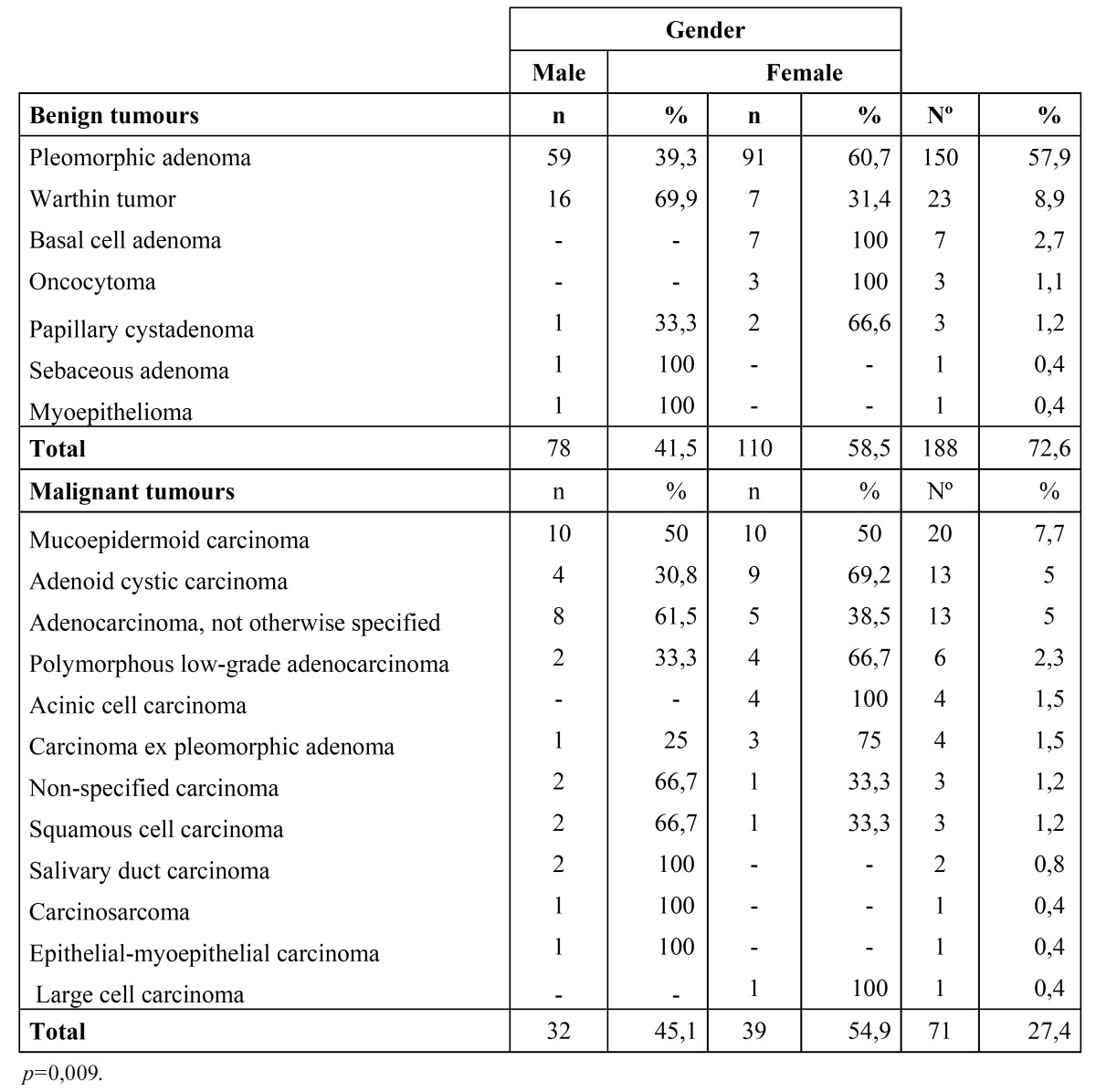
Primary epithelial tumours by gender.

There was a wide age distribution for the salivary gland tumours in this study, with a range of 4 to 87 years (average 53.3 ± 19 years). The average age for men and women was 55 ± 18.4 and 52.1 ± 19.3 years respectively. The average age at which benign tumours were diagnosed was of 50.7 ± 19.2 years while malignant tumours were diagnosed at an average age of 60.2 ± 16.9 years. The differences between age of appearance of malignant and benign tumours were statistically significant (*p*=0.03).

- Primary non-epithelial tumors:

Nineteen tumours (6.8%) of the total of 279 tumours were non-epithelial. Of the non-epithelial tumours, 11 cases (58%) were lymphomas, and four cases were lipomas (21%) ( Table 3).

**Table 3:**
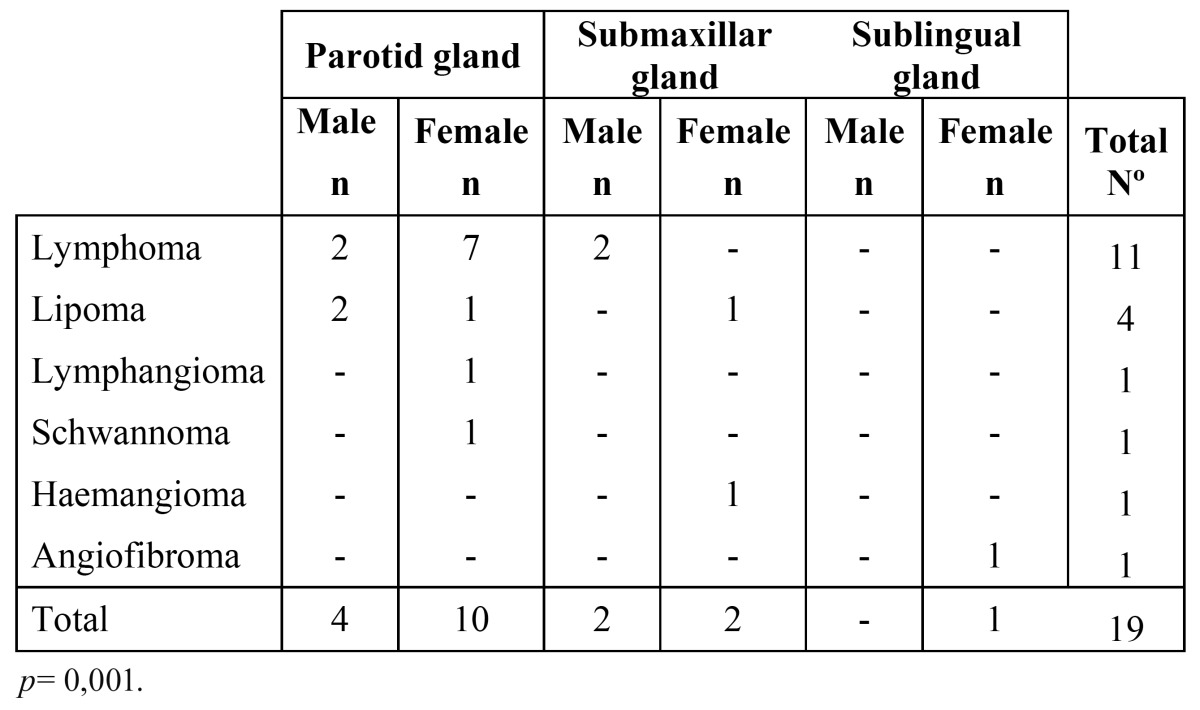
Non-epithelial tumours.

The distribution of the non-epithelial tumours according to gender and location is shown in  Table 3. The majority of the tumours (n=14) were located in the parotid gland, which was statistically significant (*p*=0.001). The non-epithelial tumours affected women more commonly, particularly the lymphomas, but these differences were not statistically significant (*p*=0.366).

- Metastasis:

There was only one case recorded as a metastasis, which accounted for 0.4% of the total number of tumours. This was found in the parotid gland of a man of 65 years and was diagnosed as metastasis of a renal clear cell adenocarcinoma.

## Discussion

This is the first study that reports on the incidence and prevalence of salivary gland tumours in the province of Valparaíso- Chile. A total of 279 salivary gland tumours were analysed in this review and together with an estimated population assigned to each hospital (1.3645.000 people) it was possible to calculate the prevalence and incidence, which were 15.4 and 2.51 cases per 100,000 respectively. It is difficult to make comparisons with other studies because these measures are not often reported in this type of series.

Seventy point three percent and 29.7% of the tumours were benign and malignant respectively. A Brazilian study of 493 salivary gland tumours (8,9) reported a distribution of 74.8% benign and 25.1% malignant tumours. Another study performed in a Brazilian population (7) reported a distribution of 67.5% and 32.5% benign and malignant neoplasms respectively. A Chinese series of 6982 salivary gland neoplasms (2), reported 68% benign and 32% malignant cases. An Iranian study of 130 cases (9) found 68.2% benign and 31.8% malignant tumours. Although these reports are from different geographical areas, they are very similar between each other and to the present review, suggesting that benign salivary gland tumours are more common than malignant tumours worldwide, with an estimated prevalence between 67 and 75% of all salivary gland neoplasms.

According to the WHO and other reports (5,8) and in agreement with the present results, female patients are overall more affected than men, although there are some studies that report higher prevalence’s in men (3,9,10,11). These differences may be explained due to geographical variations of the populations.

It is reported that mesenchimal tumours account for 1.9% to 5% (12) of all salivary gland tumours. Takahama *et al*. (12) reported in their series of 600 cases that 95% of the tumours were of epithelial origin, while the remaining 5% were of non-epithelial origin. The majority of the cases in the present review were of epithelial origin (92.8%) with only 19 cases (6.8%) of non-epithelial origin, which is slightly different than the other reports.

Tian *et al*. (2) and Li *et al*. (10) reported in their series that the majority of primary epithelial salivary gland neoplasms were located in major salivary glands, especially the parotid gland. In the present study 77.22% of the primary epithelial tumours compromised major salivary gland with a marked predilection for the parotid gland, making it the most affected gland. The minor salivary glands together were the second more common location, representing 22.78% of the cases. The submandibular gland was the third most frequent location. This distribution corresponds well to previous reports (2,3,10,13). Some authors (1) have reported the submandibular gland to be the second most affected gland followed by the minor salivary glands. These difference may be due the fact that these authors do not reported the prevalence of tumours in minor salivary gland as a whole group, reporting the prevalence per location (palatal, labial, buccal, etc.). There were no primary epithelial tumours affecting the sublingual gland in the present series confirming the low frequency of these tumours in this location.

The majority of the epithelial tumours were benign (72.6%), similar to that which has been reported by other authors (3,8,9). Forty nine point two percent of the tumours in the minor salivary glands were located in the palate. Sixteen of them were malignant and 13 were benign. While this suggests that malignant neoplasms are more common than benign neoplasms in the minor salivary glands of the palate, the difference was not statistically significant. These findings are consistent with those reported in the literature (1,7,9,13-19).

When analyzing the epithelial tumours by histological type, the most prevalent neoplasm was the pleomorphic adenoma (57.9%). The most frequent location of this lesion was the parotid, submandibular and minor palatal salivary glands respectively, similar to that which has been reported in other studies (1,6-9,13,15,19-22). Warthin tumor was the second most common lesion, corresponding to 8.2% of all epithelial tumours. Similar studies reported this tumour to be the third or fourth most common type, being outnumbered by the mucoepidermoid carcinoma and/or the adenoid cystic carcinoma. All reports agreed that the most common location is the parotid gland (1,6,7,9,13,15,16,19-22).

The mucoepidermoid carcinoma represented the third most common tumour and was the most frequent malignant neoplasm, followed by the adenoid cystic carcinoma and adenocarcinoma not otherwise specified. These results are similar to previous series (2,3,6,7,10,13,15). Some of these studies reported that the mucoepidermoid carcinoma was the most common malignant tumour and others suggested that the adenoid cystic carcinoma was the most common. These differences may be explained due to regional variations regarding to age, gender and race.

Most of the pleomorphic adenomas were found in females, which was statistically significant (*p*= 0.009). Similar results have also been found by other authors (7,8). Warthin tumour was found more frequently in males, which has also been reported by other series (2,3,7-9) In relation to mucoepidermoid and adenoid cystic cell carcinoma, the present and other published series have not been able to established a gender predilection for either lesion.

The average age of presentation for the primary epithelial tumours was 53.3 years, which varied considerably between the benign and malignant tumours. The benign neoplasms occurred at an average age of 50.7, whereas the malignant neoplasms occurred at an average age of 60.6. This corresponds to previous studies which have estimated that malignant neoplasms occur approximately a decade later when compared to benign tumours (1,7,9,13-19).

In relation to the non-epithelial neoplasms, the parotid gland was the most commonly affected gland (73.7%) followed by the submandibular (21%) and the sublingual (5.3%) glands respectively. This is similar to the findings reported by Cho *et al*. (11). In their study, the parotid gland was the most commonly affected gland with 83.3% of the cases, followed by the submandibular gland with 17.7% of the cases. The authors reported no tumours in the sublingual gland. In the present series, lymphoma corresponded to the most prevalent histologic type, with 58% of the cases followed by lipomas with 21% of the cases. According to the gender distribution of these neoplasms there was a M:F ratio of 1:1.38 which is very similar to the one reported by Cho *et al*. (11), which was 1:1.25. The average age for this group of patients was 53.2 years.

Most lymphomas were located in the parotid gland, which was statistically significant (*p*=0,035). There was a tendency to affect women more than men, but this was not statistically significant. The average age of presentation for this neoplasm was 65.36 years. Considering the low prevalence of lymphomas in the head and neck region and given the location, age and gender of these patients, it is possible that could have be affected by Sjögren’s syndrome. It has been reported that lymphomas arise in 4-10% of patients with Sjögren’s Syndrome (22). Unfortunately that information was not available from the medical records.

## Conclusion

Salivary gland tumours are uncommon neoplasms that usually arise in the parotid gland showing some predilection for females. Benign tumours are by far more common than malignant tumours. Pleomorphic adenoma and mucoepidermoid carcinoma were the most common benign and malignant tumours reported in this series respectively. Almost half of the tumours arising from minor salivary glands were malignant, so special care must be taken when these glands are affected.
